# On Applications of Polymer Materials—Adsorption, Catalysis, and Degradation

**DOI:** 10.3390/ma19153225

**Published:** 2026-07-29

**Authors:** Magdalena Sobiesiak

**Affiliations:** Department of Polymer Chemistry, Institute of Chemical Sciences, Faculty of Chemistry, Maria Curie-Sklodowska University, Maria Curie-Skłodowskiej 3sq., 20-031 Lublin, Poland; magdalena.sobiesiak@mail.umcs.pl

## 1. The Special Issue Short Resume

This Special Issue was dedicated to the applications of polymers and related materials in adsorption and catalysis, as well as their degradation processes. It embraced a total of 12 published scientific articles (including one review) which, within a period of 3.5 years since the launch of the Special Issue, garnered a total of 35,070 views and 139 citations, highlighting its significance and academic impact. Considering the subject areas of the published articles, the most popular topics related to:

- Preparation of copolymers and composites (five articles);
- Sustainable and biodegradable materials (four articles);
- Processing and modification of polymers (four articles);
- Adsorption (four articles);
- Degradation (two articles);
- Medical purposes (two articles).

However, this Special Issue also accommodated more niche topics, such as research on elastomers used in soft robotics devices and the application of an artificial neural network to evaluate the factors with the strongest influence on the dye sorption process in composite materials.

A wide spectrum of possible implementations of polymers confirms they constitute a significant group of extensively used materials, with development prospects that remain open. For this reason, the Special Issue “Applications of Polymer Materials—Adsorption, Catalysis, and Degradation” is an important source of knowledge in the field of modern polymer applications and confirms the growing interest and impact of this area of research.

## 2. Introduction

In recent years, remarkable progress in the field of polymer materials has been observed. In particular, this applies to their utilization in adsorption and catalysis processes. Advances in polymer chemistry and materials engineering have enabled the development of highly functional systems with tunable porosity [1,2], tailored physicochemical properties and surface chemistry [3,4], and enhanced thermal stability [5,6,7]. These innovations have significantly improved the performance of polymer-based adsorbents in environmental remediation, especially in wastewater treatment, where efficiency, selectivity, and reusability are critical [8,9,10,11]. At the same time, to meet the demands of green chemistry, the direction of research has shifted towards sustainable and environmentally friendly materials, with increased emphasis on biopolymers and hybrid systems derived from renewable and natural-origin sources [12,13,14,15,16,17,18,19,20].

Despite making significant strides, there are still areas we need to explore further. The fundamental relationships between polymer structure, morphology, and performance are not yet fully understood, particularly for complex and multicomponent systems [21,22,23]. Moreover, adsorption mechanisms under realistic operating conditions where multiple pollutants and matrix effects coexist require deeper investigation [19,24,25]. Additional challenges include the limited scalability of advanced materials, insufficient integration of predictive modeling tools, and the lack of comprehensive lifecycle strategies that ensure recyclability and environmental compatibility [15,26,27,28,29].

## 3. Contributions of the Special Issue

Among all the published articles, the largest number focused on the synthesis of porous polymers, copolymers, and composites as sorption materials used in separation and purification techniques, where the underlying processes are adsorption or adsorption-and-desorption [30]. Removal of some model compounds like dyes or ionic polymers from aqueous solutions allows not only to show the application potential of the tested materials, but also to understand the course of the observed phenomena [31]. Insightful data analysis leads to an explanation of relationships between the composition of adsorbents and their properties by correlating physical structure, e.g., crosslinking density or porosity, and surface chemistry with adsorption performance or mechanical strength [32]. Here, the role of internal organization of the polymer network in its thermal stability and degradation, particularly in porous and crosslinked systems such as copolymers, cannot be omitted. These studies help understand the ways of degradation and highlight how chemical composition and network architecture influence thermal resistance [33].

The analytical process can also be enhanced by integrating artificial neural networks. This modeling-based approach has identified practical ways to link experimental data with predictive tools, thereby contributing to a more effective evaluation of the factors (such as pH and ionic strength) influencing the efficiency of the adsorption [34].

Another key area is the ongoing transition toward sustainable materials. Studying biodegradable polymers, natural fillers, and cheap hybrid adsorbents brings us closer to environmentally compatible alternatives and greener technologies. Comparison of PLA and PLA–diatomaceous earth composites for 3D printing shows how the control of microstructure—specifically porosity reduction and structural rearrangement—leads to significant improvements in mechanical performance, directly linking processing conditions with material functionality [35].

On the other hand, environmental conditions affect polymers, causing their aging and deterioration of properties. A thorough understanding of these processes is of vital importance to the engineering field. Industrial stressors and exposure factors—including distilled water, hydraulic oil, cooling oil, and UV rays—affect the mechanical properties of polymers. Corrosion and aging processes impair the mechanical strength of structural components made of plastics. In the case of elastomers, such processes result in reduced flexibility and an increased tendency to rupture. If the elastomers serve as components of, e.g., soft robots, knowledge of their resistance to degradation caused by external factors makes it possible to estimate the service life of the entire device. In the publication by Rusu et al. [36], it appears that UV radiation has the most destructive impact on tensile strength and elasticity of the tested silicone rubber, whereas the liquid media caused relatively minor corrosive changes.

Beyond that, polymer materials are widely used in modern medicine. Medical applications of polymers fall into several categories. One of them is surgery and implantology, where they are used to manufacture heart valves, vascular prostheses, surgical sutures, tissue adhesives, and artificial joints (e.g., hip sockets) [37]. In ophthalmology, polymers are the foundation for the production of contact lenses and intraocular implants. Meanwhile, in dentistry, PMMA (poly(methyl methacrylate)) and other polyesters serve as the base for composite fillings, dentures, and orthodontic appliances [38,39].

The use of polymers as drug delivery systems has enabled the controlled and targeted release of medications, which allows patients to experience fewer side effects, e.g., during chemotherapy [40].

Biodegradable and bioresorbable materials—such as poly(glycolic acid) or poly(lactic acid)—are crucial in regenerative medicine. They serve as scaffolds for cells, as well as dissolvable sutures and orthopedic screws. Their use eliminates the need for follow-up surgeries to remove implants [41]. Moreover, modern hydrogels and hydrocolloids create an optimal environment for wound healing (e.g., for burns or ulcers), leading to their use as wound-dressing materials. The latter is addressed in a study by Dechojarassri et al. [42], where the authors evaluated and compared the cytotoxicity of multilayered hyaluronic acid/chitosan/bacterial cellulose-based and alginate/chitosan/bacterial cellulose-based membranes, prepared via the layer-by-layer method. It was demonstrated that membranes containing hyaluronic acid supported higher fibroblast viability than their alginate-containing counterparts. These results indicate that hyaluronic acid enhances the biocompatibility of medical dressing materials, contributing to the development of new, advanced wound coverings or more complex wound management systems.

## 4. Future Prospects

Ultimately, the real value of this Special Issue is how it points the way forward for future research.

First, in the future, research should prioritize advanced characterization techniques, particularly in situ and operando methods, to better understand the dynamics of processes occurring at the interface between a polymer and the environment. These approaches are key to linking observed performance with the underlying molecular mechanisms.

Second, because of the promising results of the initial applications of artificial neural networks and machine learning, this direction should be expanded. Future efforts could focus on building comprehensive datasets and coupling experimental data with predictive algorithms to accelerate the discovery and optimization of new materials.

Third, sustainability strategies must evolve toward a holistic lifecycle approach, incorporating not only biodegradable materials but also recycling, regeneration, and reuse. Designing polymer systems that maintain high performance over multiple cycles while remaining environmentally friendly will be critical for real applications.

Finally, scalability and industrial implementation remain major challenges. Future studies should include pilot-scale validation, long-term stability testing, and techno-economic analyses to ensure that promising laboratory-scale materials can be successfully translated into practical solutions.

Further advancement in the field will depend on interdisciplinary collaboration, integrating polymer chemistry with biology, medicine, and environmental science, process engineering, or other areas, for example, the space sector.

## 5. Conclusions

“Applications of Polymer Materials—Adsorption, Catalysis, and Degradation” Special Issue not only consolidates recent achievements in polymer materials research but also provides a clear outlook on future challenges and opportunities. By charting new pathways, it provides a strong foundation for future research into innovative, sustainable, and high-performance polymers.

## Data Availability

Not applicable.
